# Exploiting metabolic inhibition to eradicate residual tumors after chemo-immunotherapy

**DOI:** 10.1186/2051-1426-2-S3-P208

**Published:** 2014-11-06

**Authors:** Tsadik Habtetsion, Gang Zhou

**Affiliations:** 1 Cancer Immunology, Inflammation and Tolerance program (CIIT), Georgia Regents University, Augusta, GA, USA

## 

Cancer cells have long been known to exhibit metabolic reprogramming which involves a shift towards glycolysis. The enhanced glycolysis of cancer cells not only impose nutrient restriction in the tumor microenvironment, but also contribute to the acidic environment which is hostile to the invading anti-tumor immune cells. Hence, targeting the glycolysis dependency of tumor cells has been exploited for therapy. The glucose anti-metabolite, 2-deoxyglucose (2DG), a competitive inhibitor of glucose transport and glucose phosphorylation by hexokinase, has been extensively tested in various animal models and clinical trials. Although 2DG as anti-cancer agent showed a limited efficacy, its combined use with other treatment modalities, including chemotherapy and radiotherapy, has shown encouraging results. In particular, it has been shown that the combination of 2DG and cytotoxic agent etoposide resulted in enhanced antitumor immune responses, suggesting the therapeutic potential of combining chemotherapy, immunotherapy and glycolysis inhibition. We set out to study how metabolic inhibition alters the tumor microenvironment and how it can be utilized to augment the efficacy of chemo-immunotherapy. We found that glycolysis inhibition alone was detrimental to adoptive T-cell therapy (ACT). However, a rational combination of chemotherapy, ACT and 2DG led to tumor eradication and long-term survival in mice. The underlying mechanism of this synergy will be discussed.

